# Recombinant Newcastle disease virus expressing P53 demonstrates promising antitumor efficiency in hepatoma model

**DOI:** 10.1186/s12929-016-0273-0

**Published:** 2016-07-28

**Authors:** Ying An, Tianyan Liu, Jinjiao He, Hongsong Wu, Rui Chen, Yunye Liu, Yunzhou Wu, Yin Bai, Xiaochen Guo, Qi Zheng, Chang Liu, Jiechao Yin, Deshan Li, Guiping Ren

**Affiliations:** 1 Biopharmaceutical Lab, College of Life Science, Northeast Agriculture University, Mucai Street 59, Xiangfang district, Harbin, People’s Republic of China; 2 Key Laboratory of Agricultural Biological Functional Gene, Northeast Agricultural University, Harbin, 150030 China

**Keywords:** Recombinant NDV, P53, Gene therapy, Anti-tumor

## Abstract

**Background:**

Numerous studies have demonstrated that the NDV-mediated gene therapy is a promising new approach for treatment of cancers. P53 plays a vital role in tumor suppression and surveillance. Therefore, we hypothesize that a recombinant NDV expressing P53 would be an ideal agent for the hepatoma therapy.

**Results:**

In the essay, the human P53 gene was incorporated into the genome of a lentogenic strain (named rNDV-P53), which did not affect viral replication kinetics and magnitude in HepG2 cells. Compared to the vehicle virus, rNDV-P53 increased cell growth suppressor ratio and early apoptosis by 2 folds, and decreased the mitochondrial membrane potential in HepG2 cells. *In vivo* studies, treatment with rNDV-P53 reduced tumor volume of tumor-bearing mice by more than 4 folds, tumor weight by more than 5 folds comparing with rNDV. The 120-day survival rate of rNDV-P53-treated mice was 75 %, survival rate of rNDV-treated mice was 12.5 %. TUNEL analysis showed a significant increase in the apoptosis rate in the tumor tissues of rNDV-P53-treated mice than that of rNDV-treated mice. Moreover, serum chemistries revealed an insignificant change of blood urea nitrogen (BUN), creatinine levels, alanine aminotransferase (ALT) and aspartate transaminase (AST) in rNDV-P53-treated group compared to normal mice, suggesting treatment with the recombinant virus was not toxic.

**Conclusion:**

rNDV-P53 is a potent candidate for carcinoma therapy especially for hepatocarcinoma.

## Background

Newcastle disease virus (NDV) is a single-strand non-segmented negative-sense RNA virus of the paramyxoviridae family [1]. Causing severe infection to multiple avian species, but is not pathogenic to humans. The genome containing 15186 nucleotides encodes six genes, including the nucleocapsid protein (NP), phosphoprotein (P), matrix protein (M), fusion protein (F), hemagglutinin neuraminidase (HN) and RNA-dependent RNA polymerase (L) [2, 3]. NDV was proposed as a promising anticancer agent [4–7], it is prone to replicate in human tumor cells and cause oncolytic effects but not in normal cells [8–10], which is a superior characteristic as a therapeutic agent for cancers compared with the other oncolytic virus. The anticancer potency of NDV depends on the ability to induce apoptosis in infected cancer cells [11–13]. However, previous studies have demonstrated that NDV induces apoptosis in tumor cells independent of the function of the P53 [14, 15].

The P53 tumor suppressor plays various roles in cell-cycle control, apoptosis, senescence, DNA repair and modulating metabolic processes [16–20]. As a transcription factor, P53 can regulate several proteins, such as pro-apoptotic Bcl-2, caspases, death receptors and so on. With the expression of these genes, cell death was induced by apoptosis [21, 22]. Meanwhile, the tumor suppressor P53 is inactivated by mutations, which can be found in about 50 % of human cancers [23, 24]. It has been proposed that the increased survival of tumor cells lacking functional P53 is due to the decreased ability of these cells to undergo apoptosis [25]. Briefly, the expression of wt-P53 gene can inhibit tumorigenesis. It is not surprising that restoring wild-type P53 activity has been used as a cancer gene therapy in both laboratory studies and clinical trials [26–29].

In the present study, we generated a recombinant NDV (named rNDV-P53) and evaluated its antitumor efficacy *in vitro* and *in vivo* tests. We showed that the virus possesses a significant oncolytic activity against hepatoma cell line HepG2 and is an effective oncolytic agent in a H22 tumor mouse model. In a conclusion, we conclude that recombinant NDV expressing P53 is a promising agent for cancer therapy.

## Methods

### Cell culture

The human hepatoma cell line of HepG2, Hep3B and the mice hepatoma cell line of H22 were supplied by northeast agricultural university biological pharmaceutical teaching and research section. The baby hamster kidney cell line of BHK21 was a generous gift from Dr. B.Moss. HepG2 and BHK21 cells were cultured in DMEM containing 10 % new-born calf serum (NCS) and 1 % penicillin/streptomycin. All cell lines were maintained at 37 °C in a 5 % CO_2_ atmosphere and 95 % humidity.

### Recombinant Newcastle disease virus

The total RNA of human peripheral blood leukocytes was prepared by using Trizol and then was transcribed into cDNA. The P53 gene was amplified by PCR using the human peripheral blood leukocytes cDNA as template and the following primers: sense 5′-ATGGAGGAGCCGCAG-3′and antisense 5′-TCAGTCTGAGTCAGGCCCTT-3′. The PCR product was purified by 1 % agarose gel electrophoresis and inserted into HpaI-MluI fragment of clone30 plasmid. The nucleotide sequence was identified by sequence analysis and compared with the reported P53 gene [GenBank: 82395019]. And then the recombinant plasmid was transiently cotransfected with helper plasmids encoding viral NP, P and L into BHK21 cells stably expressing T7 RNA polymerase using lipofectamine 2000. The virus was rescued and amplified by inoculation of the supernatant from the transfected cells into the allantoic cavity of specific-pathogen free chicken embryos.

### Determination of virus growth

Virus growth was determined in HepG2 cells culture. HepG2 cells in 6-well plates were infected with recombinant virus at 37 °C in DMEM supplemented with 10 % new-born calf serum and 1 % penicillin/streptomycin in a 5 % CO_2_ atmosphere. Cells with supernatants were frozen at the time indicated i.e. 24, 48, 72, 96 h post-infection. After repeated freezing and thawing 3 times, the cells with supernatants were collected. The concentration of virus was determined by end-point titration on chicken embryo fibroblast cells and was expressed as mean log_10_ 50 % tissue culture infective dose (TCID_50_) per ml. Finally, according to the virus titer in different time a growth curve was drawn.

### Determination of exogenous P53 protein expression by Western blotting

HepG2 and Hep3B cells (5 × 10^6^ cells) were infected with rNDV-P53 at 1 MOI. After 24 h incubation, cells were collected and washed twice with cold PBS by centrifugation at 500 × g for 5 min at 4 °C. The pellet was resuspended in lysis buffer supplemented with proteases inhibitor and the supernatant was stored at -20 °C. For western blotting analysis, samples were separated by 10 % sodium dodecylsulfate-poly acrylamide gel electrophoresis (SDS-PAGE), and transferred to a nitrocellulose membrane. The blot was visualized by chemiluminescence and autoradiography using X-ray film. Mouse anti- human P53 polyclonal antibody (DO-1) was obtained from Santa Cruz Biotechnology Inc., CA, USA. A protein marker (New England Biolabs, Beverly, MA, USA) was run for each gel to identify P53.

### Cell viability assay

A short-term microculture tetrazolium (MTT) assay was used to quantify cell viability. Approximately 2 × 10^4^ HepG2 cells were plated into 96-well plates in complete medium and allowed to attach for 24 h. Subsequently, the cells infected with rNDV-P53, rNDV at 0.01 MOI, 0.1 MOI, 1 MOI, and 10 MOI in triplicate. After 48 h of incubation, 20 μl MTT solutions (5 mg/ml in sterile phosphate-buffered saline) were added to the cell. 4 h later, the MTT solution in the wells was discarded, then 150 μl dimethyl sulfoxide (DMSO) was added. The absorption at 490 nm (OD490) was measured on a microplate reader. The cell viability was converted and expressed as the percentage of the control. The cells without any treatment were used as negative control.

$$ \mathrm{Inhibition}\ \mathrm{rate}\ \% = \left(\mathrm{control}\ \mathrm{group}\ \mathrm{O}\mathrm{D}\ \hbox{-}\ \mathrm{treatment}\ \mathrm{group}\ \mathrm{O}\mathrm{D}\right)\ /\mathrm{control}\ \mathrm{group}\ \mathrm{O}\mathrm{D}\times 100\% $$

### Annexin V –FITC and PI binding assay

Approximately 2 × 10^4^ HepG2 cells were plated into 6-well plates in complete medium and allowed to attach for 24 h. Subsequently, the cell infected with rNDV-P53, rNDV at 0.1 MOI, 1 MOI, and 10 MOI in triplicate. After 48 h of incubation, cells (0.5 ~ 1x10^6^) were digested with 0.25 % trypsin, then resuspended by adding 200 μl Binding Buffer. Then the cells were incubated away from light with 5 μl Annexin V labelled FITC 30 min at room temperature. At last, after being mixed with 5 μl PI and 300 μl Binding Buffer and reacted 5 ~ 15 min away from light, the cells were quantitatively detected by flow cytometry (usually within 1 h), at the same time a tube cells without AnnexinV-FITC and PI served as a negative control. Cells were analyzed in a flow cytometry using FL1 (530 nm) bandpass filters of FACS Calibur (Bectom Dickinson, USA), and data were analyzed by the CellQuest software (BD, USA).

### Measurement of mitochondrial membrane potential

Changes in mitochondrial transmembrane potential (ΔΨM) were measured by using 5,5′,6,6′-tetrachloro-1,1′,3,3′-tetra-ethylbenzimidazolylcarbocyanine iodide (JC-1) as a probe. HepG2 cells were plated in six-well plates at 1 × 10^5^ cells/well and infected by the recombinant virus rNDV-P53 and rNDV at 1 MOI. After 12 or 24 h of incubation, cells were digested with 0.25 % trypsin, then resuspended in 1 ml DMEM containing JC-1 (20 μg/ml). Then cells were incubated for 30 min in dark at room temperature. Cells were washed twice with PBS, re-suspended in 0.5 ml PBS, and immediately analyzed in a flow cytometry using FL1 (590 nm) band pass filters of FACS Calibur (Bectom Dickinson, USA).

### *In vivo* evaluation of tumor growth

All procedures involving animals followed the guidelines issued by National Institute of Health and the Institutional Animal Care and Use Committee of Northeast Agriculture University (approval number: SCXK-2011-0004).

Six-week-old male ICR mice (with a mean weight of 18 g) were housed in a pathogen-free environment and implanted subcutaneously with 2 × 106 H22 cells into the right groin. When the tumor size reached about 50 mm3, the animals were randomly divided into three groups. Then the tumor-bearing mice were intratumoral injection inoculated with a single injection of 1 × 107 pfu of rNDV-P53 or rNDV in a 200 μl volume and 200 μl of PBS were injected instead of the virus, which represented as control. Tumor growth was observed every 2 days for 18 days by measuring the two dimensional longest axis (L) and shortest axis (W) with a caliper. The tumor volume was calculated by using the following formula: volume in mm3 = 4/3 × π × (L/2 × W/2 × W/2). According to the institutional protocols, the mice were euthanized when the tumors reached 18 mm in diameter. On day 18, all mice from the control group and 6 mice from each treatment group were sacrificed and their tumors were excised. The tumors were weighted and immersed in 4 % paraformaldehyde. Then all sections were cut at 4 mm thick from buffered formalin-fixed, paraffin-embedded tumors. After deparaffinization, sections were stained with haematoxylin/eosin (H&E) and prepared for TUNEL assay (terminal deosynucleotidy transferase-mediated dUTP nick and labeling assay). In addition, serum was prepared from whole blood for determination of serum concentrations of AST, ALT, BUN, and creatinine. The remaining mice in each treatment group were observed for 120 days with measurement of tumor volume. This was disease-free 120-day survival rate.

### Statistical analysis

All the data were obtained from at least three independent experiments. The data are expressed as mean values ± SD and were compared using Student′s t test. Statistical significance was defined as a P value < 0.05.

## Results

### Recovery of the recombinant virus rNDV-P53

**Fig. 1:**
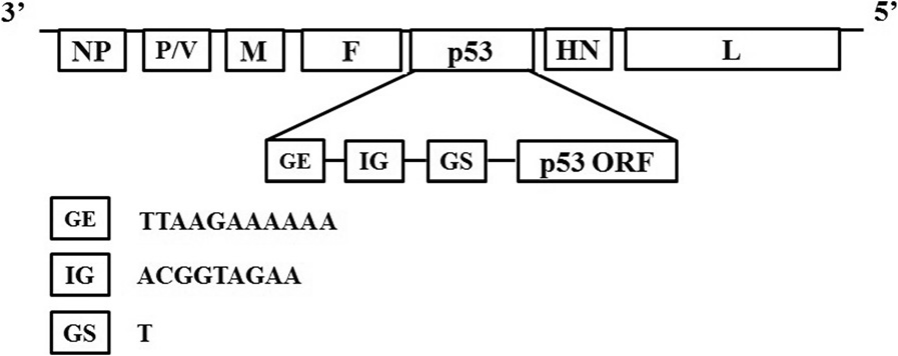
Construction of rNDV-P53. Diagram showing insertion of the P53 gene into the NDV genome at the position between the F and HN genes

The human wild-type P53 gene was inserted into the newly created Hpa I and Mlu I sites between the F and HN genes of the NDV genome (Fig. 1). Then we generated the recombinant virus rNDV-P53. The incorporated plasmid was transfected into BHK-21 cells. Then the supernatant of the transfected monolayers was injected into 10-day-old SPF embryonated chicken eggs. After 3 days, the allantoic fluid was harvested and analyzed in a HA test. The high titers of rNDV-P53 suggested the successful generation of the recombinant NDV virus.

### Growth and exogenous P53 protein expression of the recombinant virus rNDV-P53

**Fig. 2:**
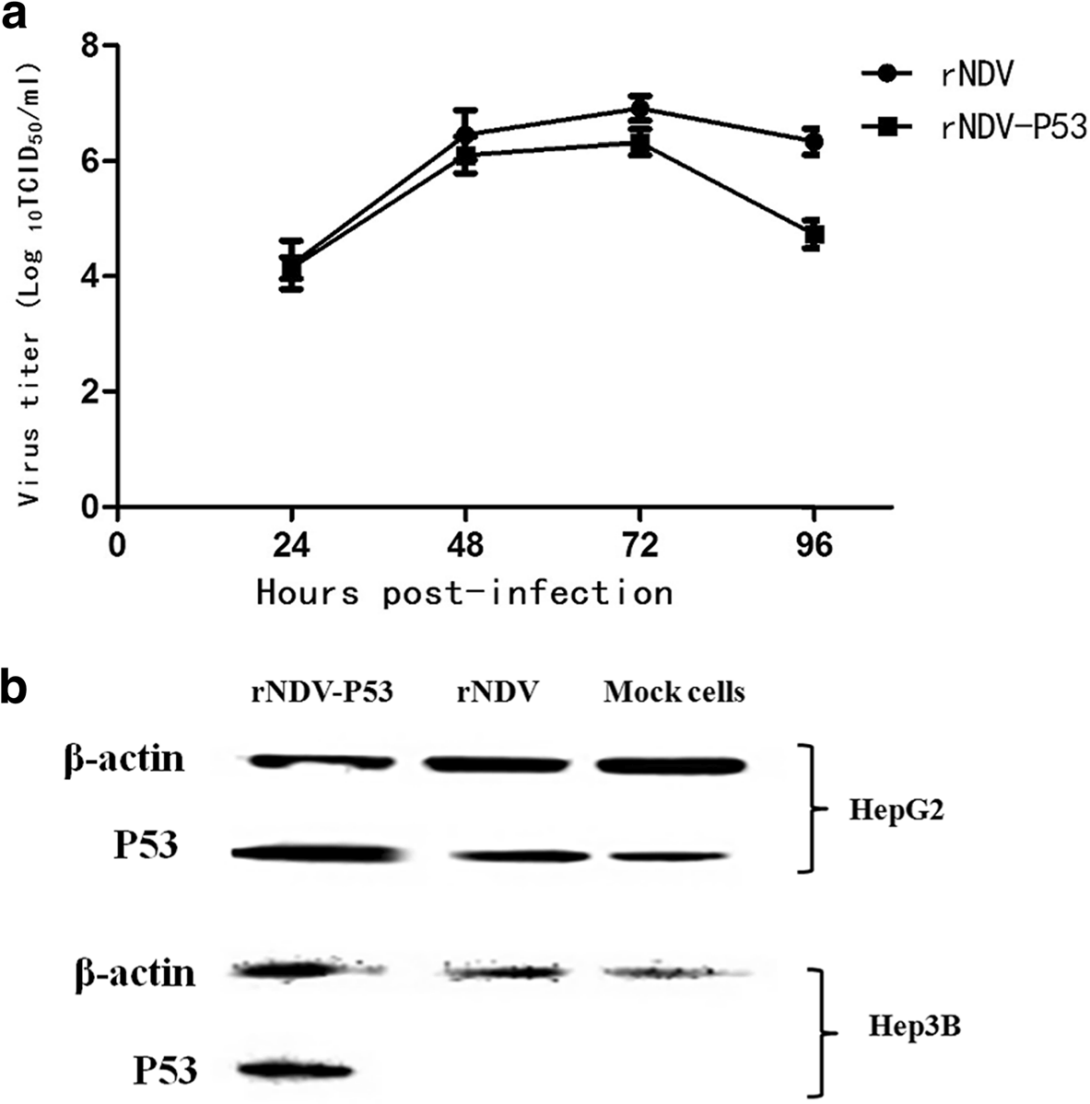
Growth curve of the recombinant virus and Exogenous P53 protein expression. **a** The virus titers in the supernatant of different time points were assessed by plating tenfold dilution on DF-1 cells and were shown as log_10_TCID_50_/ml. **b** The β-actin was used as control

In order to verify the recombinant virus rNDV-P53 remains stable proliferation in tumor cells, HepG2 cells were infected with rNDV-P53 at MOI of 1 and the supernatant of them was harvested at different time points. Then the viral titers in the supernatants were determined in triplicate. As shown in Fig. 2a, compared with the parental virus, the recombinant virus rNDV-P53 has no any distinctions in the kinetics and magnitude of replication while the production of rNDV-P53 was slightly delayed. To investigate the expression of P53 protein, HepG2 and Hep3B cells were treated with rNDV-P53 at MOI of 1. Western blotting analysis (Fig. 2b) showed that the P53 expression increased in the tumor cells infected with rNDV-P53.

### Recombinant virus rNDV-P53 suppresses tumor cell growth *in vitro*

**Fig. 3:**
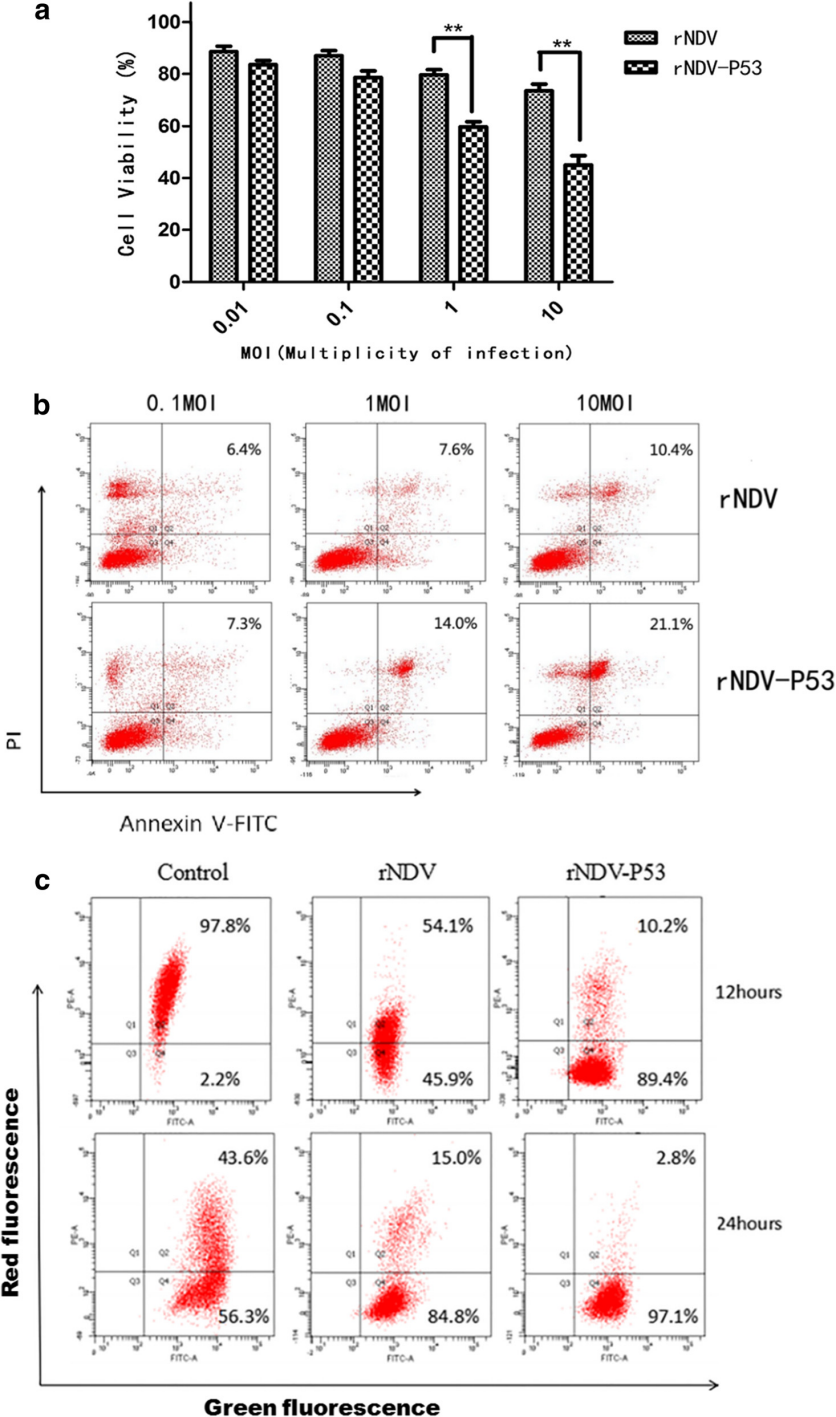
The recombinant virus rNDV-P53 induces cell death by apoptosis in human tumor cells HepG2. **a** Cytotoxicity of rNDV-P53 in tumor cells. HepG2 cell were infected with rNDV or rNDV-P53 virus for 24 h. Cell viability was determined by MTT assays (mean ± SD). Replicates were averaged, and the error bars represent the SD of three independent experiments. **b** The cells were infected with the rNDV or rNDV-P53 in different dosages. Cell apoptosis was detected by Annexin V-FITC/PI Apoptosis Detection Kit according to the manufacturer′s instructions and followed by flow cytometry. **c** After 12 h or 24 h of virus infection, HepG2 cells were stained with JC-1 and analyzed by flow cytometry

The direct cytotoxic activity of the recombinant virus rNDV-P53 was tested in HepG2 tumor cells in this study. As shown in Fig. 3a, rNDV-P53 induced cell death in a dose-dependent manner during 24 hour′s treatment in HepG2 cells. When the cells were infected with rNDV-P53 or rNDV of 10 MOI for 24 h, the viability of HepG2 cells dramatically decreased to 45.03 % ± 3.6 %, 73.67 % ± 2.51 %, respectively. In the meanwhile, the cell growth suppressor ratio induced by rNDV-P53 is up to 1.7 times of rNDV (MOI = 1). Collectively, these data demonstrate that the recombinant virus rNDV-P53 is superior to parent virus in suppressing the growth of HepG2 cells.

To determine the sensitivity of tumor cell to recombinant virus-induced apoptosis, HepG2 cells were treated with recombinant virus rNDV-P53 for 24 h, and the percent of apoptotic cells was determined by Annexin V-FITC/PI assay. Apoptosis assay showed that after virus infection the tumor cell death by apoptosis was occurred markedly. The results showed that apoptosis percentages of rNDV group and rNDV-P53 were 10.4 % and 21.1 % respectively, which indicated that virus rNDV-P53 were more effective in inducing apoptosis in the HepG2 cells (Fig. 3b). In line with the observation of cell death, rNDV-P53 induced apoptosis in a dose-dependent manner.

To explore the molecular mechanisms by which virus induced cell death, the mitochondrial membrane potential of HepG2 cells infected with virus was assayed by using the fluorescent dye JC-1. JC-1 accumulates in mitochondria as aggregates, resulting in red fluorescence in non-apoptotic cells. The monomeric form, which fluoresces green, is found in apoptotic cells. As shown in Fig. 3c, the green fluorescence of JC-1 increased in HepG2 cells treated with virus over the time course. Both rNDV and rNDV-P53 reduced the mitochondrial membrane potential compared with the PBS group, but the effect of rNDV was not as potent as rNDV-P53. These results indicate that the recombinant virus can effectively reduce the mitochondrial membrane potential, which is the key in cell apoptosis.

These data suggest that the insertion of P53 gene improved the ability of NDV inducing tumor cell apoptosis.

### The recombinant virus rNDV-P53 suppresses tumor growth in mice

**Fig. 4:**
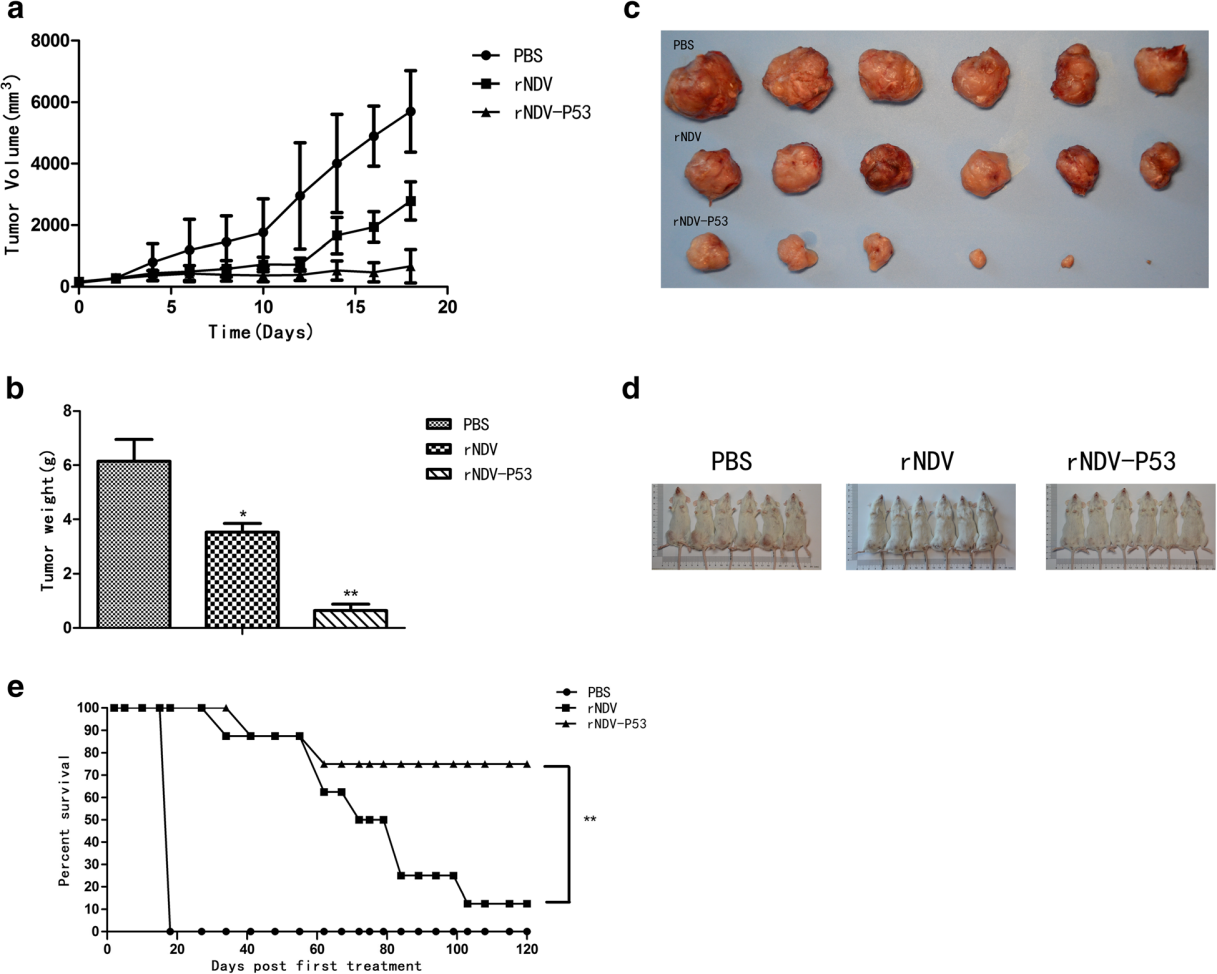
The recombinant virus rNDV-P53 suppresses the growth of H22 xenografts in mice**.** A xenograft H22 animal model was established in mice. When the tumor size reached to about 50 mm^3^ (about 7 days post-inoculation), the tumors were injected with 1 × 10^7^ pfu of rNDV or rNDV-P53 virus (day 0). The PBS was used as control. The injections were repeated every 2 days during experimental period. Mice were killed when the tumors reached 18 mm in length. On day 18, all mice from the control group and 6 mice from each treatment group were sacrificed and their tumors were excised. The remaining 8 mice in each treatment group were observed for 120 days with measurement of tumor volume. **a** The tumor growth was observed by measuring the tumor size every 2 days for 18 days. **b** The weight of the excised tumors of different groups. **c** The excised tumors of different groups. **d** Before necropsy, the tumor in the right groin of different groups mice. **e** The percent survival of tumor-bearing mice treated with PBS, rNDV, and rNDV-P53 in the 120 days (**p < 0.01; *p < 0.05)

The mice H22 hepatocellular carcinoma model was employed in our study for examining the tumor suppressing efficacy of the recombinant virus rNDV-P53 *in vivo*. When the tumor size reached about 50 mm^3^, the animals were divided into three groups. The mice were intratumorally treated with 1 × 10^7^ pfu of rNDV, rNDV-P53 or PBS every other day for nine injections. The tumor growth was observed by measuring the tumor size every other day for a total of 18 days. After nine injections, all animals in the PBS group were sacrificed due to excessive tumor burden. The average tumor size for the PBS-control group was 5702.93 ± 540.25 mm^3^, while 666.96 ± 222.62 mm^3^ noted for the rNDV-P53 group. For the group treated with rNDV, the average tumor sizes were 2785.45 ± 254.30 mm^3^ (Fig. 4a). Moreover, the average tumor weight of PBS-control group was 6.14 ± 0.81 g. For the group treated with rNDV and rNDV-P53, the average tumor weight was 3.52 ± 0.33 g and 0.64 ± 0.24 g, respectively (Fig. 4b). During necropsy, tumors excised from the control group were fairly large in size and tumors isolated from the rNDV-P53 group were generally small (Fig. 4c, d). Taken together, these data demonstrate that the recombinant virus rNDV-P53 have a very significant effect in tumor inhibition comparing with rNDV.

In this study, the survival time course of the tumor-bearing mice was also recorded. Over the 120 days (Fig. 4e), remaining 7/8 mice in the rNDV group developed significant size and needed to be sacrificed. While only 2/8 mice in the rNDV-P53 group developed tumors that required to be sacrificed. The remaining mice in each group either completely cleared or had persistent pigmented focus that did not change in size. The overall survival of the mice in the long-term study was 0/8 for control group, 6/8 for rNDV-P53 group and 1/8 for rNDV group.

**Fig. 5:**
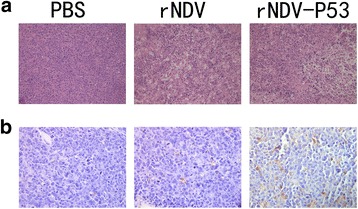
Representative sections of H&E-stained mouse tumors and TUNEL-stained mouse tumors dissected on day 18. **a** Mouse tumor by H&E-stained. **b** Apoptotic cells in the tumor tissues were visualized by TUNEL assay. Brown colored cells represent apoptotic cells. PBS was used as control (magnification, 400X)

As shown in Fig. 5a, H&E staining tumor sections show tumors derived from rNDV-P53 demonstrated a stronger suppression to cellar viability than rNDV and PBS group. Then we evaluated the cytotoxic effect of the recombinant virus rNDV-P53 on tumor issue by TUNEL assay. Compared with a control group, we found a significant increase in the apoptosis rate of the rNDV-P53 group (Fig. 5b). These data indicate that rNDV-P53 induces apoptosis, consequently inhibiting the growth of tumor tissue.

**Fig. 6:**
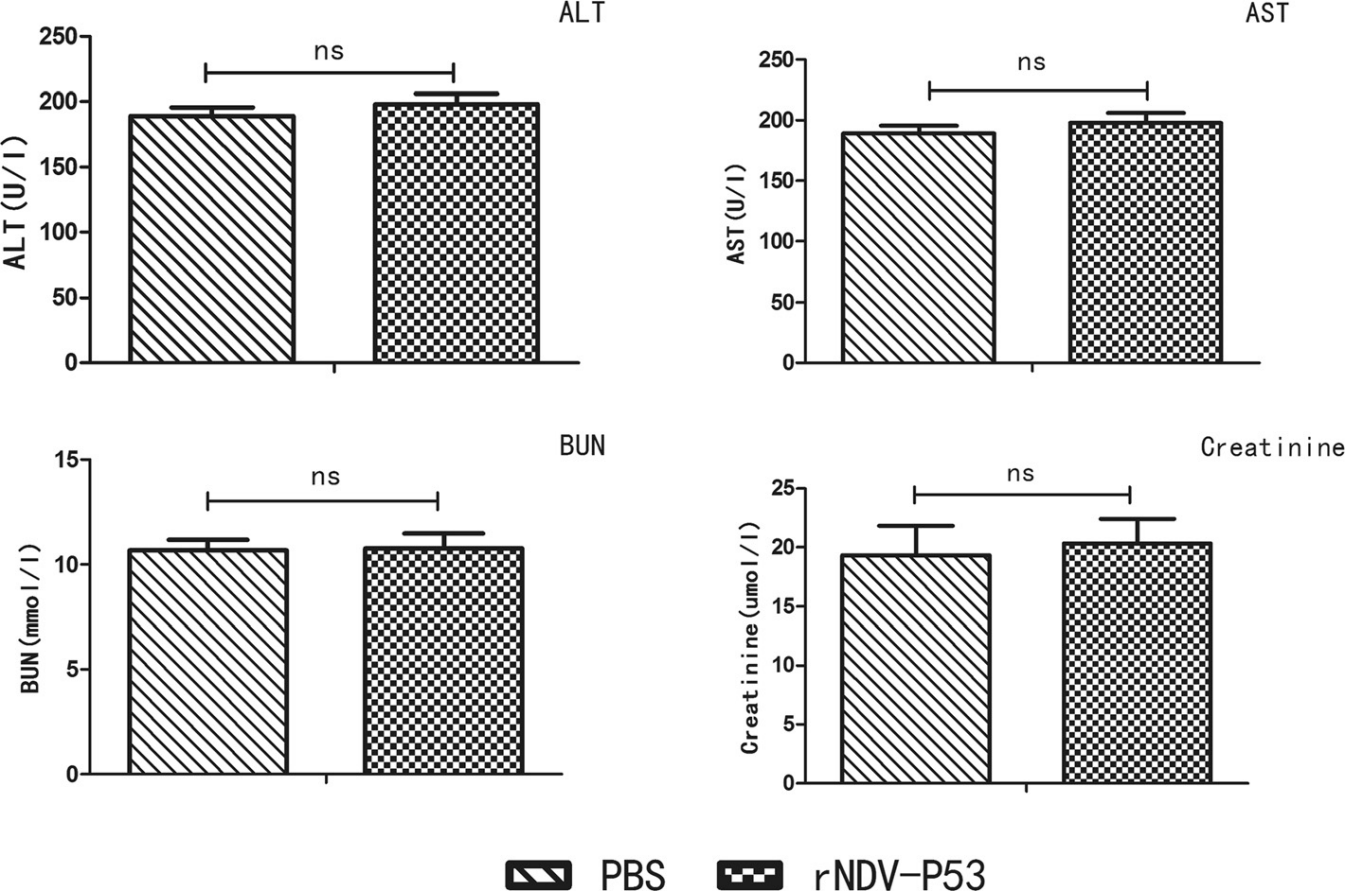
The serum biochemistry data of the experimental mice after injection of rNDV-P53. After ten injections, the blood of mice was drawn for measurement of serum chemistries. Values are expressed as mean ± SD. ALT, alanine aminotransferase; AST, aspartate transaminase; BUN, blood urea nitrogen; PBS, phosphate-buffered saline; ns, no significant

In order to assess the safety and reliability of the recombinant virus administration *in vivo*, a toxicity study was performed in mice. After ten injections, the blood of mice injected with PBS and rNDV-P53 was collected. Serum chemistries revealed insignificant changes in BUN, creatinine levels, AST and ALT (Fig. 6). According to the results, we concluded the systemic effects of the rNDV-P53 treatment at mice were similar to the PBS treatment. Briefly, the recombinant virus is safe.

## Discussion

Newcastle disease virus (NDV) has a number of advantages as an attractive candidate for viral vector for cancer therapy. First of all, NDV can selectively replicate in tumor cells and cause lysis of cancerous cells while sparing normal cells [30–32]. Secondly, NDV mediates antitumor effects by inducing apoptosis in cancer cells. Multiple subsequent studies confirmed that the apoptosis plays a dominant role in NDV-induced cell death, and the apoptosis inducing effect does not depend on the presence of functional P53 protein in the infected tumor cells [33–36]. Lastly, NDV is a bird virus without pathogenicity in humans, in extreme cases of virus infections, humans only have mild conjunctivitis, laryngitis and flu-like symptoms. In addition, as a viral vector, NDV does not integrate into the human genome [37–40]. These facts contribute to its safety.

The tumor suppressor P53 is a stress-activate transcription factor that regulates several genes with a broad range of functions including DNA repair, apoptosis, cell cycle arrest, metabolism and senescence [20]. In approximately 50 % of human tumors, P53 gene is mutated and losses its function [41]. It is the reason why P53 has emerged as a promising target of gene therapy techniques [42]. P53 gene therapy is aim to prevent tumor formation or lead to tumor regression by restoring the activity of P53. So a strategy has been provided to eliminate tumor cells and induce apoptosis by reintroducing wild-type P53 into tumor cells. In the early 1990s, scientists delivered P53 into tumor cells to induce apoptosis and growth inhibition [43]. Later studies demonstrated the effectiveness of P53 gene therapy, for instance the recombinant adenovirus expressing human P53 achieved good curative effect on malignant liver cancer, gastric cancer, bladder cancer and cervical cancer [44–47].

Due to the superiority of Newcastle disease virus as a vector for cancer therapy and the effectiveness of P53 gene therapy, we constructed the recombinant virus rNDV-P53 using reverse genetics. As we expected, the kinetics and magnitude of replication of the recombinant NDV didn′t have a significant change, the P53 expression increased in the tumor cells infected with rNDV-P53. In addition, we have shown that compared with the vehicle virus, rNDV-P53 significantly inhibited HepG2 cells growth. We hypothesized that the ability of rNDV-P53 in inducing cell death was enhanced by the inserted gene P53. To confirm this conjecture, we tested this hypothesis in cancer cell lines HepG2. When the cells were infected with virus of 10 MOI for 24 h, the early apoptosis rate induced by rNDV-P53 was 21.1 % and the vehicle virus was 10.4 %. Apoptosis analysis showed that rNDV-P53 strengthens pro-apoptotic activity of virus. It is consistent with the previously report that restoring wild-type P53 activity leads to apoptosis in cancer cells [48]^.^ Previous investigation suggests that the apoptosis induced by P53 is accompanied by the decrease of mitochondrial membrane potential [49]. Using the fluorescent dye JC-1, we observed a further loss of membrane potential causing by the rNDV-P53 compared with its parental stain (Fig. 3c). Based on the encouraging data *in vitro*, we investigated the therapeutic efficacy of rNDV-P53 in H22-bearing mice. At the end of the experiment, the tumor weights and volumes significantly decreased in mice treated with rNDV-P53 compared with those treated with rNDV or PBS. The average tumor volume in rNDV-P53 group was 666 mm^3^ verses over 2000 mm^3^ in rNDV or PBS group. The average tumor weight in rNDV-P53 group was 0.64 ± 0.24 g verses 3.52 ± 0.33 g in rNDV group and 6.14 ± 0.81 g in PBS group, respectively. These results indicate that rNDV-P53 can effectively inhibit tumor growth. In addition, TUNEL experimental results showed that the rNDV-P53 has a stronger ability to induce apoptosis in tumor tissue than rNDV. rNDV-P53 significantly increased the survival rate of mouse liver cancer model. At the end of the 120 days survival experiment, the overall survival of the untreated model mice was 0/8 verse 6/8 for the rNDV-P53 group and 1/8 for the rNDV group. In order to assess potential toxicities associated with recombinant virus *in vivo*, a toxicity study was performed. AST and ALT are the important indexes in evaluating liver function, the blood urea nitrogen and creatinine are the important indexes in evaluating renal function. We have detected the BUN, creatinine, AST, and ALT. Results showed that these parameters were in the normal range, indicating that rNDV-P53 is relatively safe for cancer therapy.

## Conclusion

In conclusion, we demonstrate a promising oncolytic agent rNDV-P53 consisting of an ideal vector and a suppressor gene P53. Our studies demonstrated that rNDV-P53 is efficacious in suppressing hepatocellular carcinoma model and has the potency to cure the mice completely. Thereby we offered a promising antitumor agent for hepatoma.

## Abbreviations

ALT, alanine aminotransferase; AST, aspartate transaminase; BUN, blood urea nitrogen; DMEM, Dulbecco's modified Eagle medium; FBS, fetal bovine serum;GE, gene end; GS, gene start; H&E, haematoxylin/eosin; JC-1, 5, 5′, 6, 6′-tetrachloro-1, 1′, 3, 3′-tetra-ethylbenzimidazolylcarbocyanine iodide; MOI, multiplicity of infection; MTT, microculture tetrazolium; NDV, Newcastle disease virus; PBS, phosphate buffer saline; PFU, plaque forming unit; TCID_50_, tissue culture infectious dose 50; TUNEL assay, terminal deosynucleotidy transferase-mediated dUTP nick and labeling assay
